# Beyond Liver Function Tests, Liver Stiffness, and Portal Pressure: Portal-Systemic Shunting as a New Endpoint for Clinical Trials of Cirrhosis and Portal Hypertension

**DOI:** 10.1016/j.gastha.2026.100935

**Published:** 2026-03-24

**Authors:** Andrew P. Keaveny, Kamran Qureshi, Stuart C. Gordon, Joanne C. Imperial, Michael P. McRae, Touraj Shokati, Gregory T. Everson

**Affiliations:** Department of Gastroenterology and Hepatology, Mayo Clinic, Jacksonville, Florida; Division of Gastroenterology and Hepatology, Saint Louis University School of Medicine, St. Louis, Missouri; Division of Gastroenterology and Hepatology, Henry Ford Health, Detroit, Michigan; School of Medicine, Wayne State University, Detroit, Michigan; College of Medicine, Michigan State University, East Lansing, Michigan; HepQuant, LLC, Denver, Colorado; Custom DX Solutions LLC, Houston, Texas; HepQuant, LLC, Denver, Colorado

A recent editorial emphasized the importance of using appropriate endpoints in clinical trials for patients with cirrhosis due to metabolic dysfunction–associated steatohepatitis (MASH).^1^ It was suggested that future trials in this population should be designed stratifying the presence of clinically significant portal hypertension (CSPH) defined by measuring the hepatic venous pressure gradient (HVPG). Because HVPG measurement is invasive and operator-dependent, noninvasive alternatives—such as combining liver stiffness measurement (LSM) with platelet count (PLT) using the Baveno criteria—have been proposed.^2^ A recent review highlighted LSM as a noninvasive surrogate for fibrosis to assess liver health for use in the clinic and clinical trials.^3^ However, LSM is also operator-dependent and may be less accurate in patients with obesity, diabetes, MASH, and advanced age.^2^

The oral cholate challenge test (OCCT) introduces a transformative approach to liver disease management by providing a simple, noninvasive, and physiologically grounded measure of hepatic function and portal-systemic shunting. Cholate clearance is hepatocyte-specific, flow-dependent, and correlates with functional acinar mass; however, endogenous cholate is unsuitable as an estimate for clearance given its fluctuations in peripheral blood concentration due to enterohepatic cycling. The solution is to administer a distinguishable form of cholate and directly measure clearance. In the OCCT, cholate labeled with deuterium (d4-cholate) is administered orally. Blood samples are collected at 20 and 60 minutes, and d4-cholate concentration is measured via liquid chromatography–tandem mass spectrometry to calculate clearance.^4^, ^5^, ^6^ Test parameters (ie, Disease Severity Index [DSI] and portal-systemic shunt fraction [SHUNT%])^4^^,^^7^ quantify severity of liver disease and predict risk for PH, large esophageal varices (LEV), and clinical outcomes.^8^, ^9^, ^10^, ^11^ Its validated performance in guiding decisions to forgo or perform upper endoscopy, predicting prognosis, and monitoring therapeutic response positions the OCCT as a practical tool for precision hepatology. By enabling earlier intervention and more personalized care, this test is poised to redefine the patient assessment in the clinic and accelerate drug development in patients with chronic liver disease.

**Figure 1:**
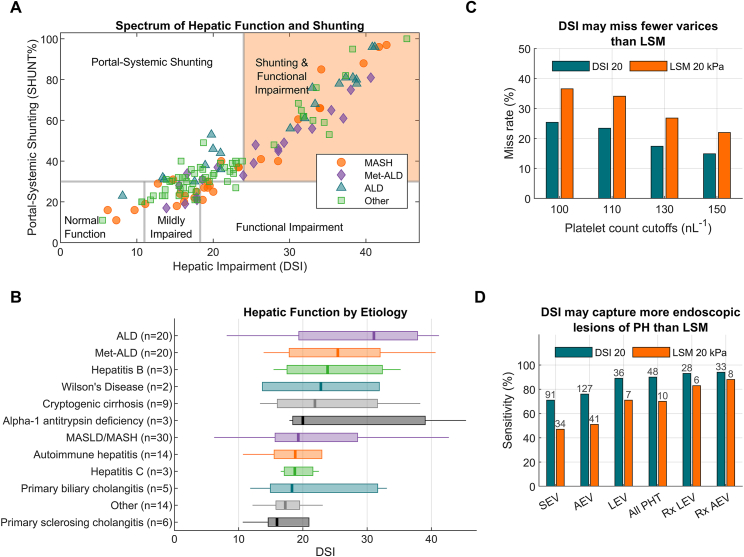
The OCCT characterized the spectrum of hepatic function and shunting in cACLD (A) and DSI by etiology (B) in the Early Access Program (n = 129). DSI from the OCCT (n = 275) compared to LSM (n = 86) for miss rate for any esophageal varices at various platelet count cutoffs used in the Baveno criteria (C) and for diagnostic sensitivity in detecting endoscopic lesions of PH (D). AEV, any esophageal varices; ALD, alcohol-associated liver disease; All PHT, all significant endoscopic lesions of PH (LEV, Rx LEV, Rx AEV, large gastric varices, varices with red wale signs, and severe portal hypertensive gastropathy); cACLD, compensated advanced chronic liver disease; DSI, disease severity index; LEV, large esophageal varices; LSM, liver stiffness measurement; MASH, metabolic dysfunction–associated steatohepatitis; MASLD, metabolic dysfunction–associated steatotic liver disease; PH, portal hypertension; Rx AEV, treated any esophageal varices; Rx LEV, banded at EGD or varices medication prescribed post-EGD; SEV, small esophageal varices.

The OCCT quantifies portal-systemic shunt from the spillover of orally administered d4-cholate into the systemic circulation as quantified by DSI and SHUNT%. Both DSI and SHUNT% have been validated for prediction of LEV and correlated to PH, CSPH, and liver-related clinical outcomes.^8^, ^9^, ^10^, ^11^ Figure 1A and B compare the quantification of liver function (DSI) and physiology (SHUNT%) in MASH vs non-MASH etiologies in a representative US clinic population.^12^ This analysis demonstrates substantial heterogeneity in liver function and physiology. Patients with highest DSI and SHUNT% values (Figure 1A, shaded area) are more likely to have LEV and experience adverse clinical outcomes. These findings support the complementary use of OCCT with other noninvasive tests in the evaluation of all patients with compensated advanced chronic liver disease.

Figure panels A and B reproduced from Gordon et al, *Liver Int Commun*, 2025, 6(4):e70027, https://doi.org/10.1002/lci2.70027, under the terms of the Creative Commons Attribution (CC BY 4.0) license. Figure panels C and D display data from Shiffman et al.^8^

In the SHUNT-V study, the diagnostic performance for LEV was compared between OCCT and LSM in a post hoc analysis.^8^^,^^11^ The analysis demonstrated that DSI missed fewer varices of any size compared with LSM across PLT thresholds from the Baveno criteria (Figure 1C). Similarly, the analysis also demonstrated that DSI 20 captured more endoscopic lesions of PH than LSM (ie, higher sensitivity for any varices, treated varices, large gastric varices, varices with red wale signs, and severe portal hypertensive gastropathy) (Figure 1D). The results were similar for DSI vs LSM comparisons of 18 vs 19 kPa, 20 vs 20 kPa, and 23 vs 25 kPa where esophagogastroduodenoscopy avoidance rates were equivalent between DSI and LSM at 38%, 43%, and 55%, respectively.

HVPG measurement is invasive and operator-dependent, and the small change in HVPG that has been suggested as meaningful clinical change for effectiveness of therapy is a high bar to achieve. Several clinical trials using HVPG as an endpoint failed to meet primary endpoint, questioning whether it was the agent itself or the choice of endpoint that led to the failed trial and wasted resources. These uncertainties also raise questions regarding the use of HVPG for patient management and as an endpoint in clinical trials.

The OCCT directly quantifies portal-systemic shunting, which parallels the rise in portal pressure, but overcomes a major limitation of HVPG measurement—the dampening of portal pressure by development of portal-systemic shunts.

A recent editorial suggested liver and spleen stiffness measurements in combination with PLT as a noninvasive alternative to hepatic venography.^1^ However, the diagnostic performance of vibration-controlled transient elastography and shear-wave elastography may be compromised in obese patients—a population that often represents a significant proportion of MASH trial participants. In the Baveno VII report, the quality of evidence supporting the use of LSM with PLT (with or without body mass index) in compensated advanced chronic liver disease due to metabolic dysfunction–associated steatotic liver disease as a predictor of CSPH was rated low (C.2, weak).

The OCCT, and specifically the DSI and SHUNT%, have been linked to clinical outcome and may be valuable endpoints in future clinical trials of cirrhosis due to MASH. Additionally, the OCCT outperformed elastography in the linkage to esophageal varices.^8^^,^^11^ In a study of treatment of PH, OCCT outperformed spleen stiffness in detecting treatment effects.^13^ Advantages of the OCCT include its noninvasive, blood-based nature; its ability to assess liver function and physiology; its simplicity of administration; and its favorable tolerability experienced by study subjects.

Using the risk for adverse clinical events model,^10^ the DSI from the OCCT estimated the potential clinical benefit of resmetirom in a 23-subject substudy of the phase 3 open-label MAESTRO-NAFLD-1 trial.^14^ The assumption was that baseline DSI and the change in DSI over the 48-week treatment trial would represent the results for the entire 122-subject cohort. In this analysis, the estimated clinical event rate after 48 weeks of resmetirom treatment mirrored the observed rate for the whole cohort.

**Figure 2:**
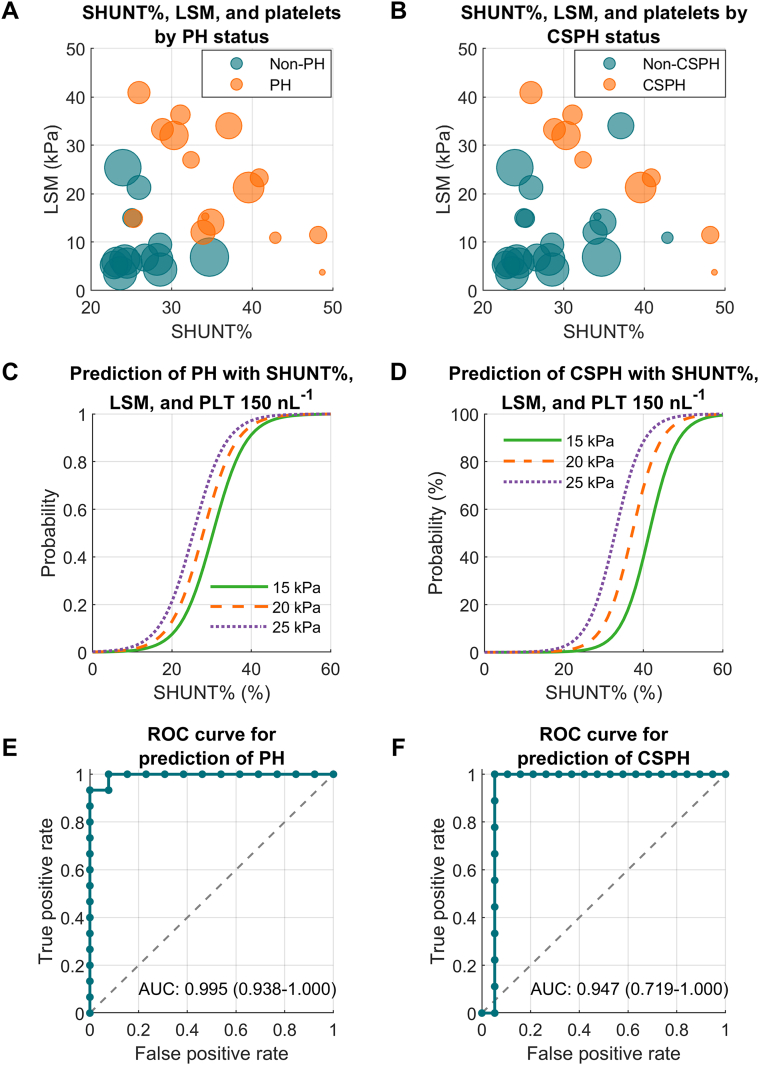
Prediction of PH and CSPH by SHUNT%, LSM, and PLT. Bubble charts show the distribution of SHUNT% and LSM by PH (A) or CSPH (B) status, with bubble size indicating PLT (min. 72 nL^−1^, max. 279 nL^−1^). Logistic regression plots show the probability of PH (C) or CSPH (D) for a given SHUNT% at 3 levels of LSM (15, 20, and 25 kPa) and 1 level of PLT (150 nL^−1^). ROC curves show the diagnostic performance of the logistic regression models including SHUNT%, LSM, and PLT for the prediction of PH (E), and CSPH (F). Data are from Wieland et al,^9^ and models are described in Hassanein et al.^15^ AUC, area under the ROC curve; CSPH, clinically significant portal hypertension; LSM, liver stiffness measurement; PH, portal hypertension; PLT, platelet count; ROC, receiver operating characteristic; SHUNT%, portal-systemic shunt fraction.

DSI and SHUNT% from the OCCT represent new, innovative endpoints for use in drug development programs. The DSI or SHUNT% alone, or in combination with other noninvasive tests, provides a more comprehensive assessment of liver health and a more accurate estimate of risk for CSPH (Figure 2)^9^^,^^15^ and liver-related outcomes. These characteristics and others warrant expanded use of the DSI and SHUNT% from the OCCT for clinical management and drug development in cirrhosis and PH due to MASH.
